# Suitability of rainwater harvesting in saline and arsenic affected areas of Bangladesh

**DOI:** 10.1016/j.heliyon.2024.e34328

**Published:** 2024-07-09

**Authors:** Md. Abdullah, Fatin Idrak, Purnima Kabir, Mohammad Amir Hossain Bhuiyan

**Affiliations:** Department of Environmental Sciences, Jahangirnagar University, Dhaka, 1342, Bangladesh

**Keywords:** Arsenic contamination, Groundwater availability, Rainwater harvesting suitability, Salinity intrusion, Spatial-temporal analysis

## Abstract

A major portion of Bangladesh is currently experiencing a scarcity of safe drinking water because of arsenic contamination, high salinity and human-induced pollution. The objectives of this study were to identify locations with a high scarcity of drinking water and suitability of harvesting rainwater. Kriging interpolation algorithms of Geographical Information System (GIS) was employed to identify the probable water scarce zones as well as suitable zones of harvesting rain water from the available data of secondary sources. Statistical methods were employed to cluster, correlate, and regress variables such as rainfall, salinity, and As. The results showed that groundwater quality in the southwestern parts of Bangladesh is saline with high concentration (>10000 μS/cm). On the other hand, the northeastern and southwestern parts of Bangladesh are also vulnerable to arsenic contamination (60 %–97 % of tubewells), compared to other regions. The rainfall zonation map, covering the years 1951–2022, indicated that the Sylhet division had the highest potential for rainfall (ranging from 2600 to 3900 mm). From this study it was demonstrated that Sylhet, Noakhali, Bhola, Barishall, Patuakhali, Bagerhat, and Khulna were identified as suitable places for sustainable rainwater harvesting (RWH). The findings of this study may play significant role towards achieving sustainable potable water supply in vulnerable zones, if they receive attention from policymakers.

## Highlights

• Bangladesh lacks safe drinking water due to arsenic and salinity.
• Spatial analysis reveals contamination-prone areas.
• Bangladesh has a wide range of suitability of rainwater harvesting in water scarce areas.
• Effective planning and implementation are essential to maximize benefits.

## 1 Introduction

The need for clean water is steadily rising in Bangladesh due to the country's dense population. For much of Bangladesh, access to potable water has been severely compromised due to saline water intrusion and arsenic pollution of groundwater. The major issue of groundwater contamination in Bangladesh, which is caused by both natural and human activity [1], affects almost 50 million people. A number of health problems, including skin diseases and cancer, can be caused by arsenic, a prominent toxic metalloid [[2], [3], [4]]. Multiple areas of Bangladesh have documented high levels of arsenic poisoning in their groundwater [[3], [4], [5]]. Reports of excessive manganese contents, which surpass both domestic and international drinking water guidelines, have also been made public [4,6]. Additionally, groundwater resources are becoming more and more salty, which is harmful to both humans and the environment. According to research conducted in the southern regions of Bangladesh, the saline concentration of water is highest in rivers, canals, deep tube wells, and ponds [[7], [8], [9]]. As a result of saltwater seepage, groundwater is no longer suitable for human consumption or agricultural usage [10,11]. Erosion, waterlogging, floods, landslides, and droughts are among the many natural disasters that plague Bangladesh and contaminate the water supply. Many lives and possessions were lost in the devastating floods that struck Bangladesh in 1974, 1984, 1987, 1988, 1998, 2004, 2007, and 2020, making it one of the world's most flood-prone nations. There has been an eightfold increase in the frequency of severe droughts in the northern half of the country in the last forty years [12]. Rainfall patterns vary across the country, with early, late, and strong downpours occurring at brief intervals [13]. Given the breadth and depth of possible climate change impacts, it is prudent to extrapolate trends in hydrologic variables into the impacts that ecosystems, humans, and infrastructure may endure. There has been an impact on Bangladesh's water supply from climate change impact [14,15]. Bangladesh is seeing an increase in the frequency of extreme weather events such as floods, cyclones, and droughts due to the accelerated pace of climate change. The availability of water sources could be affected by these frequent events, making them more difficult to reach.

Bangladesh, a densely populated nation with few freshwater resources, is worried about the quantity and quality of its drinking water. Thus, gathering drinkable water by rainwater harvesting can be regarded as a sustainable and eco-friendly practice. Bangladesh has plenty of opportunities to collect rainwater because it receives enough rain, particularly during the monsoon season. Rain that falls on land surfaces, rooftops, and catchment areas is gathered and directed into storage facilities including underground reservoirs, cisterns, and tanks [16]. When assessing whether rainwater collection is feasible, it's crucial to consider the quantity and distribution of rainfall in a given area. In order to guarantee clean and accessible water, rainwater collection systems, suitable storage tanks, and purifying technology are essential [17]. Affordability, water consumption, and population density are socioeconomic issues that may affect rainwater collection's feasibility. However, the effectiveness of this approach depends on the acceptance and adoption of the local community. Rainwater can naturally collect on rooftops, in catchments, and on other impervious surfaces. It reduces dependency on potentially dangerous or contaminated groundwater sources by offering a consistent and dispersed source of clean water. It has come to light as a potential tactic for enhancing water security and reducing the effects of groundwater contamination at the same time [18].

Replenishing aquifers with surplus rainfall aids in addressing the decline of groundwater levels and mitigates the intrusion of saltwater in coastal regions. The nation regularly utilizes groundwater for both home and agricultural applications. On the other hand, groundwater in numerous areas is polluted with arsenic and salinity, rendering it unfit for consumption. Collecting rainwater in this case is a very promising alternate water supply. Rainwater can serve as an alternative source and have a substantial positive impact on regions with elevated levels of groundwater pollution. Rainwater collecting has positive environmental impacts by alleviating the strain on natural water resources and diminishing the demand for energy-intensive water purification processes. It can mitigate dependence on groundwater, provide a dependable water supply during droughts, and minimize the risk of flooding. Rainfall is also seen as a comparatively secure and enduring alternative due to its overall lack of toxins and affordability [19]. Implementing this solution can result in cost savings on water bills and infrastructure maintenance, making it economically viable. The potential quantity of rainfall collected is determined by the size and composition of the catchment area, such as rooftops or open spaces. Expanding the area from which rainwater is collected and improving the efficiency of the devices used can optimize the amount of rainwater that can be collected. Rainwater harvesting devices can mitigate the risk of flooding caused by excessive rainfall during monsoon seasons [20]. Multiple research [[2], [3], [4], [5], [6],^8^,^9^] has been undertaken on salinity contamination, pollution, and rainfall harvesting at the local level [12,13,15,[18], [19], [20], [21]]. However, these studies lack integration with each other. Therefore, it is crucial to combine the areas at risk of pollution with the expected amount of rainfall to assess a secure and environmentally-friendly zone for collecting drinkable water sources. This study is unique because it identifies the risk zones for groundwater at a nationwide level and investigates the prospective locations for sustainable rainwater harvesting. It achieves this by integrating pollution and rainwater harvesting, which has not been done by any other scientific community. The primary objective of this study is to conduct a thorough evaluation of the necessity for rainwater harvesting in Bangladesh, considering its implications for addressing concerns related to groundwater quality and water scarcity. The main research aims of this study are as follows: (a) to identify the areas in the country that are sensitive to groundwater contamination due to salinity and arsenic; (b) to analyze the prospective zones in Bangladesh for collecting rainwater based on long-term rainfall data.

## 2 Study area

**Fig. 1:**
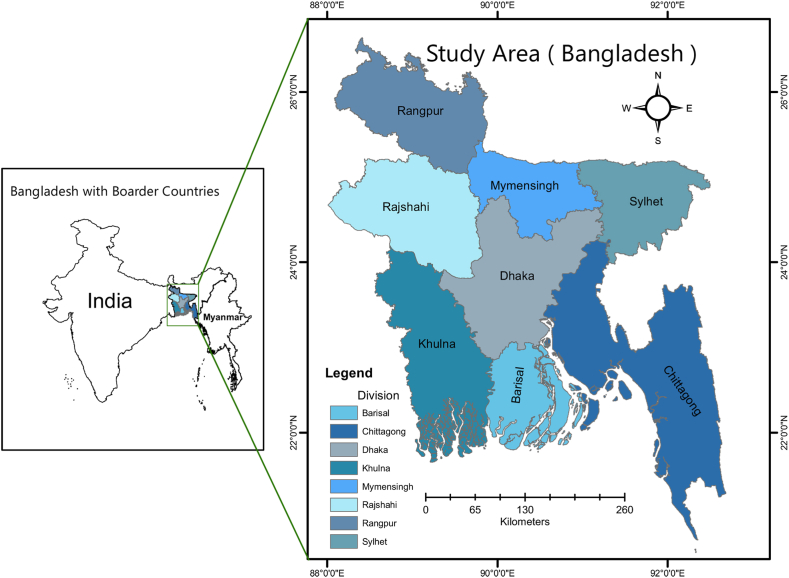
Location map of the study area.

The study encompasses the entirety of Bangladesh, a country renowned for housing the largest delta in the world, referred to as the Bengal delta [Fig. 1]. The country is in the northeastern portion of India, with its limits ranging from 20° N to 27° N and 88° E to 93° E. Approximately 80 % of the country's land area was encompassed by flood plains [22]. Bangladesh is renowned for its high population density, which renders it exceptionally vulnerable to water scarcity and associated difficulties. The nation encountered both monsoonal rainfall and drought, rendering it an ideal site for assessing the feasibility of rainwater harvesting. The study focused on several locations of Bangladesh, considering factors such as rainfall patterns, topography, population density, and existing water infrastructure. The study focused only on water-related concerns, including groundwater contamination from arsenic, groundwater salinity, and climate change. The northern highlands of the country consist of mountainous terrain. Bangladesh's southern portion, known as the Chittagong Hill Tracts, and its northeastern part, Sylhet, both included high terrain. Bangladesh experiences four distinct seasons based on its climate: (1) a dry winter season from December to February; (2) a hot summer season preceding the monsoon from March to May; (3) a wet monsoon season from June to September; and (4) a post-monsoon autumn season from October to November [23].

## 3 Methods and methodology

### 3.1 Data types and sources

The following Table 1 contains some of the data types and sources that will be used in the study on rainwater harvesting suitability in Bangladesh.

**Table 1:** Data types & sources.

Data Type	Source	Year
Precipitation	ERA5-Land monthly averaged data set: Climate Data Store (CDS), Copernicus Climate Change Service (C3S) [14]	1951 to 2022
Groundwater Arsenic	International Groundwater Resources Institute (IGRAC) [15]	2020
Groundwater salinity	British Geological Survey (BGS) [16]	1998–1999
	Department of Public Health Engineering (DPHE) [16]	

#### 3.1.1 Rainfall

The dataset “ERA5-Land Monthly Aggregated-ECMWF Climate Reanalysis” contains comprehensive data on both large-scale and localised rainfall. The variable used to record this data is referred to as the “total rainfall sum”. The time period ranged from 1950 to the present, enabling researchers to analyze long-term patterns in rainfall. The device accumulated rainfall and offered worldwide coverage, encompassing regions with elevated terrain. Users can access this variable in Earth Engine by filtering the total rainfall sum band and exporting subsets of data [24].

#### 3.1.2 Groundwater arsenic

The GGIS supplied data on arsenic contamination in groundwater in Bangladesh, which was crucial for evaluating the nation's concerns on the quality of its groundwater. We used spatial data, specifically latitude and longitude coordinates, in addition to arsenic concentration data that provided a comprehensive account of the arsenic levels in groundwater at each sampling station. The data provided valuable insights into the areas that needed further attention in order to enhance the quality of the country's groundwater [25].

#### 3.1.3 Groundwater salinity

Dissolved mineral concentrations and groundwater arsenic levels were sourced from the BGS. In 1998 and 1999, BGS and the Gov's DPHE collaborated to conduct a nationwide investigation of groundwater contaminants in 3534 wells across Bangladesh [26]. Groundwater sodium, potassium, calcium, and magnesium concentrations were measured using inductively coupled plasma-atomic emission spectrometry, whereas arsenic levels were evaluated using hydrogen generation-atomic fluorescence spectrometry [26]. For this purpose, we interpolated the salt, potassium, calcium, and magnesium concentrations in Bangladesh's groundwater using the BGS-DPHE data in conjunction with the Kriging feature in ArcGIS. For every year when the BDHS cluster geocodes were utilized to extract the components, our study treated the concentrations of these elements as mineral exposure in drinking water.

### 3.2 Google Earth Engine

GEE is a robust online platform that was launched in 2010. It provides users with access to a vast amount of satellite imagery and geographical data, together with cloud computing infrastructure and research tools. Researchers and scholars can utilize GEE to examine and track the Earth's ecology and human society on a global level. GEE has various applications such as monitoring deforestation, mapping land use change, assessing crop health, tracking glacier retreat, and managing natural disasters. The use of GIS is crucial in enhancing our comprehension of the Earth and its intricate systems, while also facilitating the development of effective remedies for environmental challenges [27].

### 3.3 JavaScript

The Code Editor for this challenge utilized JavaScript as its programming language. The decision was made based on the language's exceptional resilience and adaptability, which are particularly advantageous when handling ERA5-Land Monthly Aggregated data. JavaScript [28] was utilized to calculate the total amount of rainfall for different locations and time periods based on historical records.

### 3.4 Programming language R

Using the programming language R, an annual rainfall study was carried out to look at annual rainfall totals and find long-term patterns and variances in annual rainfall. The temporal study includes the following: noteworthy year-to-year fluctuations, seasonal summation, naming of the wet and dry months, correlation, temporal trend analysis, and descriptive statistics [29].

### 3.5 Trend analysis

The non-parametric Man-Kendall test was used to analyze trends in the current investigation. Using this statistical technique, the temporal trends and regional variation of hydroclimatic data are being investigated. A non-parametric test is favored over a parametric one since it can circumvent the problem brought on by data skew [30]. The Man-Kendall test is advised when evaluating many stations in a single study [31]. Mann (1945) created the Mann-Kendall test as a non-parametric trend detection tool, while Kendall (1975) offered the test statistic distribution for assessing non-linear trends and turning points.

#### 3.5.1 Man-Kendall trend analysis

A trend in time series data may be detected using this approach. An outlier is eliminated using this non-parametric test. This method provides three categories of data.

1. A measurement of the slope's monotonicity is the Kendall Tau, sometimes called the Kendall rank correlation coefficient.
2. Kendall's Tau is a statistical measure that fluctuates between −1 and 1, indicating a positive trend and a negative trend, respectively.
3. The entire slope of the time series is assessed by the Sen slope. Between the series' points, this slope represents the average of all the slopes that have been determined.

The significance denotes the moment at which the absence of a trend is deemed plausible. An indication that the trend is statistically significant is a p-value less than 0.05. Their relative rankings (R1, R2, R3 …., Rn) (from 1 to n) are substituted for the n time series data (X1, X2, X3, …., Xn).

$$\begin{array}{c} S=\sum \{\sum sgn\;\left(R_{i}-R_{j}\right)\;\}\\ \begin{array}{cc} i=1⎩j=i+1 & ⎭ \end{array} \end{array}$$

where,

For x > 0, sgn (x) = 1; for x = 0, sgn (x) = 0; and for x < 0, sgn (x) = −1.

If the null hypothesis (Ho) is true, then S has a roughly normal distribution with μ = 0.

$$\delta =n\frac{\left(n-1\right)\left(2n+5\right)}{18}$$

The Mann Kendall z-statistic is written as

$$Z=\frac{\left|S\right|}{\sigma^{.0.5}}$$

A positive S value denotes an increasing tendency, and vice versa. For 90, 95, and 99 % probability levels, the critical test statistic values are 1.645, 1.97, and 2.57 for the various significance levels for the observations. These tests are applied to temperature and rainfall data in order to identify patterns and quantify change over time and geography.

### 3.6 Spatial analysis

$$\text{Rainwater}\;\text{harvesting}\;\text{suitability}\;\text{of}\;a\;\text{point}\;\left(\text{Raster}\;\text{calculation}\right)=\frac{A*50}{X}+\frac{B*25}{Y}+\frac{C*25}{Z}$$

A = Rainfall value of the point.

B= Arsenic value of the point.

C= Salinity value of the point.

X = Highest rainfall value of Bangladesh.

Y= Highest arsenic value of Bangladesh.

Z = Highest salinity value of Bangladesh.

**Fig. 2:**
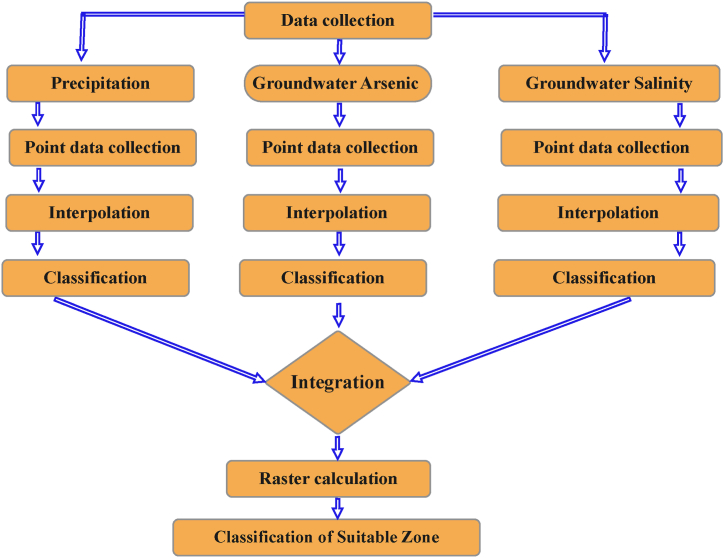
Work flow diagram of spatial analysis. Fig. 2 depicts the workflow followed for the spatial analysis conducted in this study. Each box represents a specific step, and arrows indicate the flow of data between steps. A more detailed description of the methodology used in each step is provided in Section 3.6 of the main text.

A spatial analysis was used in the study to identify places with high rainwater harvesting suitability, where rainwater harvesting can provide a consistent and secure source of water. For the analysis, GEE and ArcGIS were used to obtain satellite imagery and geographic data. To ascertain whether rainwater harvesting was suitable and effective in different Bangladeshi locations, spatial research was required. This study employed concentration data and geographic coordinates from the GGIS to analyses salinity and arsenic in groundwater in Bangladesh. Finding places with high and low rainfall, analyzing historical rainfall data to build maps of rainfall patterns, and merging several datasets were all necessary steps in the process of identifying rainwater harvesting zones. Additionally, the research employed spatial analytical methods, such as suitability modelling, to identify and priorities sites with high suitability for rainwater collection. According to Fig. 2, maps were made and geographical visualization tools (ArcGIS) were used to construct graphs, maps, and spatial statistics to visually depict the results of the spatial study [[32], [33], [34]].

## 4 Results and discussion

### 4.1 Temporal analysis of rainfall data

#### 4.1.1 Monthly variation of rainfall

**Fig. 3:**
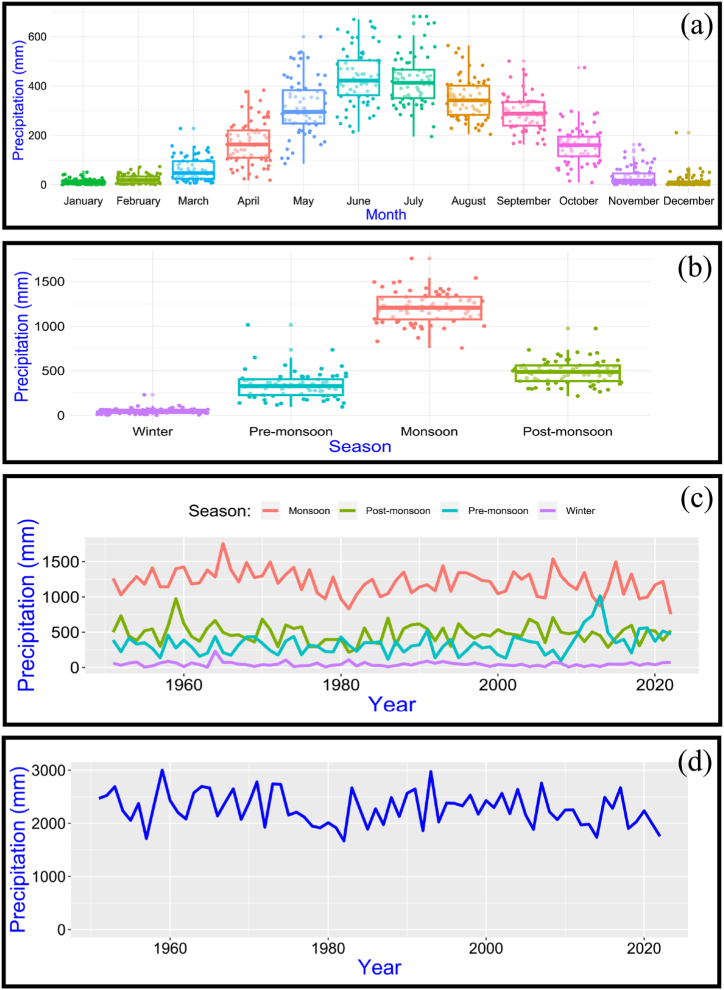
(a) Monthly Boxplot, (b) Seasonal Boxplot, (c) Seasonal precipitation in years, and (d) Annual precipitation plot of Bangladesh. Fig. 3 presents various visualizations of rainfall data for Bangladesh. Panel (a) shows boxplots for monthly rainfall distribution, highlighting the median, quartiles, and outliers. Panel (b) displays seasonal boxplots, comparing rainfall patterns across different seasons. Panel (c) depicts the seasonal rainfall variation over several years. Finally, panel (d) illustrates the annual rainfall distribution across Bangladesh.

Through the course of time, the analysis of monthly rainfall patterns has led to a comprehensive comprehension of the distribution of rainfall in Bangladesh. These findings are crucial for assessing the optimal months for rainwater collection and determining the feasibility of using rainwater harvesting as a substitute for conventional water sources. The results of this analysis contribute to the comprehensive evaluation of the study area's capacity for rainwater harvesting. Fig. 3(a) illustrates the rainfall pattern in Bangladesh during the month of January for the previous 72 years. This repetitive pattern illustrates the variations in rainfall during the month of January. Remarkably, the mean yearly rainfall in January amounts to 11.22 mm. The comparatively lower value of this statistic indicates a consistent although declining trend in rainfall levels over the month of January. This dataset provides specific details about the climate in Bangladesh for the month of January, as well as information about weather patterns in the surrounding region. Comprehending and predicting climate patterns in the region is of utmost importance.

February saw a decrease in rainfall compared to January in Bangladesh. With an average of 21.7703 mm, February has had the second-worst rainfall in the past 72 years. This data lends credence to January's claim that February saw less rainfall than usual. The winter climate of the area was better understood because to this research. January and February saw very little rainfall, suggesting that the weather was dry. There is a little more rainfall in March compared to winter in Bangladesh [Fig. 3(a)]. March rainfall has averaged 60.0417 mm during the previous 72 years. Rainfall is expected to increase, possibly indicating the beginning of the rainy season. The weather was very unpredictable in March, with the highest rainfall recorded at 227.20 mm. Six millimeters was the smallest quantity of rainfall recorded. The weather in Bangladesh can vary greatly in March, as shown by this severe difference.

Significant variations can be seen in Fig. 3(a), which shows 72 years' worth of April rainfall in Bangladesh. Rainfall in April is heavy (average 168.3708 mm), which has an impact on the ecosystems in the area. A total of 314.3542 mm of rainfall fell in April. Given that this high number is more than twice as high as usual, there could be wide variations in the amount of rainfall this month. By contrast, the lowest amount of rainfall recorded in April was 18.30 mm. Studies conducted over a 72-year period show that Bangladesh experiences greater rainfall in May. The average amount of rainfall for this month is 314.3542 mm. The weather is altered by the rise of rainfall. 599.40 mm was the most rain that fell in May. This extremely high score suggests that this month's rainfall will vary significantly. On the other hand, May's lowest recorded rainfall of 85.10 mm illustrates the variety of weather patterns.

Bangladesh experiences much greater rainfall in June than in other months [Fig. 3(a)], with an average monthly rainfall of 439.59 mm. The most rain that fell in June was 702.20 mm. This high number suggests that there might be some rain this month. Even still, the lowest June rainfall total 214 mm was still noteworthy. Bangladesh's wettest month is still July, with an average rainfall of 421.25 mm [Fig. 3(a)]. With 681.70 mm of rainfall, July had the most in 72 years. This intense flood underlines the unpredictability of the weather and the potential for significant monsoon rainfall. July was the wettest month in contrast. Fig. 3(a) shows Bangladesh's rainfall patterns during a 72-year period in the month of August. August continues to have the greatest rainfall in the extensive dataset, having recorded the most of it. With 203.90 mm of rainfall, the lowest amount was much less than the average. 347.57 mm of rainfall falls on average yearly, which is a little less than in June and July. It is possible that the monsoon pattern has altered throughout this period.

In Bangladesh, the month of September usually experiences a decrease in rainfall compared to earlier months [Fig. 3(a)]. The average amount of rainfall recorded during a period of 72 years is 291.0375 mm. The rainfall levels decline during the monsoon changes, potentially suggesting the conclusion of the wet season. The month with the highest amount of rainfall recorded a total of 500.70 mm. Conversely, the minimum reported rainfall was 163 mm, suggesting a period of drought. Accurate data on September rainfall is vital for agricultural and water resource management. In October, Bangladesh experiences an average rainfall of 159.0375 mm, as indicated in Fig. 3(a), signaling the conclusion of the monsoon season. The maximum amount of rainfall recorded in October was 474.30 mm. This intense rainfall likely had a significant impact on the surrounding environment. By contrast, the minimum amount of rainfall recorded in October was 8.20 mm. The significant disparity highlights the variation in rainfall between these two months, which is approximately twenty times less than the amount of rainfall in September.

Fig. 3(a) illustrates the amount of rainfall in the month of November spanning from 1950 to 2021. Throughout this period, the level of rainfall fluctuated. The average rainfall in November is 32.6250 mm. The year with the highest amount of rainfall recorded was 162.6 mm, and the year with the lowest amount was only 0.2 mm. The figure indicates substantial variability in the amount of rainfall during the month of November. The fluctuations in November rainfall observed over the past 72 years illustrate the dynamic nature of the region's weather and climate. Fig. 3(a) illustrates the December rainfall data spanning from 1951 to 2021. Multiple rainfall patterns and variations occurred throughout this time. The yearly fluctuations in December rainfall demonstrate the dynamic nature of regional weather patterns. The mean December rainfall for the past 72 years was 13.3432 mm. The minimum amount of rainfall recorded was 0.10 mm, while the maximum amount was 210.90 mm. The yearly fluctuations in December rainfall demonstrate the dynamic nature of regional weather patterns.

#### 4.1.2 Seasonal variation of rainfall

This section gives the results of an analysis of seasonal rainfall patterns in Bangladesh throughout the research period. Comprehending the fluctuations in rainfall patterns throughout the year is essential for assessing the availability of water resources and the appropriateness of rainwater collection as a source of water. The research primarily focuses on the pre-monsoon, winter, monsoon, and post-monsoon seasons. Based on Fig. 3(b) and (c), the rainfall levels in Bangladesh have exhibited significant patterns throughout the winter season over a span of 72 years, from 1951 to 2022. The event commences in the year 1951, with a rainfall range of 60.6 mm. Between 1951 and 1963, the data displayed a clear and noticeable zigzag pattern. Over the course of these years, the amount of rainfall varied from 60.6 mm to 2.9 mm, indicating that there were years with both higher and lower levels of rainfall. Remarkably, the recorded amount of rainfall during this entire period never exceeded 86.4 mm, which was the highest level observed in 1985. In 1963, the rainfall was at its lowest point, with only 2.9 mm. Nevertheless, in 1964, there was a significant deviation from this pattern as rainfall unexpectedly and substantially increased, reaching an astonishing 230.2 mm. 1964 holds the record for the most amount of rainfall in the whole dataset. The rainfall levels varied between the years 1965 and 2022. The annual rainfall levels exhibited variability. Nevertheless, none of these years exceeded the record levels of rainfall set in 1964. In the period after 1964, the year that experienced the highest amount of rainfall was 1983, with a total of 109.2 mm. The data shown depicts a chronological account of rainfall in Bangladesh, characterized by an alternating pattern initially, followed by a notable surge in 1964 and subsequent years, with varying quantities of rainfall.

The mean rainfall computed throughout the 72-year duration is around 46.34 mm. Fig. 3(b) and (c) depict the seasonal rainfall patterns in Bangladesh over a span of 72 years prior to the monsoon season. Except for the years 1958, 1964, 1968, 1969, 2008, 2019, 2020, 2021, and 2022, the amount of rainfall in these years falls within the range of 200–400 mm. The annual rainfall has above 400 mm in each of these years. There was a slight upward trend in 2011, 2012, and 2013, culminating in a record high of 1015.7 mm, the most in 72 years. In 2011, the amount of rainfall was 646.8 mm, however in 2012, it increased to 732.9 mm. The rainfall amounts were less than 200 mm in the years 1957, 1962, 1966, 1972, 1975, 1986, 1992, 1995, 2001, and 2008. In 2008, the recorded rainfall was at its minimum, with 95.1 mm. Bangladesh has experienced an average pre-monsoon rainfall of 335.3681 mm over the past 72 years.

Fig. 3(b) and (c) can be employed to examine the 72-year monsoon rainfall patterns in Bangladesh as well. The amount of rainfall has remained within a range of 600 mm and has not shown a reduction of more than 200 mm over the past 72 years. During the period from 1951 to 1965, the amount of rainfall varied between 228.4 and 446.6 mm. In 1966, the amount of rainfall in Bangladesh reached 563.7 mm, which was the highest recorded total for the monsoon season in the past 72 years. The rainfall pattern exhibited a zigzag trajectory between 1967 and 2009, indicating fluctuations in rainfall over the course of 42 years. The greatest recorded rainfall was 551.4 mm in 2010, followed by the second highest amount of 534 mm in 2012. Moreover, the rainfall varied from 219.1 mm to 406.5 mm for the period from 2013 to 2022. In 2019, the recorded rainfall reached its minimum at 219.1 mm. Over the past 72 years, Bangladesh has experienced an average monsoon season rainfall of 349.70 mm.

Fig. 3(b) and (c) depict the rainfall levels in Bangladesh during the period after the monsoon season for the previous 72 years. The amount of rainfall ranges from 200 mm to over 1000 mm. Upon doing an analysis of the rainfall patterns in Bangladesh over the initial nine-year period from 1951 to 1959, it was determined that the maximum recorded rainfall throughout this timeframe was 976 mm in the year 1951. Except for the highest recorded rainfall amounts, the range of rainfall depicted in the chart falls between 216.1 mm and 732.4 mm. In 1981, the recorded rainfall reached its minimum with a total of 216.1 mm. It is imperative that most of the rainfall occurs within the range of 400–600 mm. Based on this data, the mean rainfall in Bangladesh during the post-monsoon period has been 482.994 mm during the past 72 years.

#### 4.1.3 Annual variation of rainfall

Fig. 3(d) depicts the cumulative amount of rainfall in Bangladesh over a span of 72 years. The data indicates a diverse range of rainfall quantities, with the bulk falling between 0.10 mm and 1668 mm. Additionally, there are multiple years where the rainfall exceeds this upper limit. The highest cumulative rainfall on record occurred in 1959, with a total of 3002 mm, signifying an unusually intense year of rainfall. In 1993, a rainfall total of 2975 mm was recorded, which is the second-highest amount ever recorded. Most of the years in the dataset exhibit total rainfall amounts between 2000 mm and 2500 mm, which is 500 mm lower than the greatest recorded rainfall in Bangladesh. This graph illustrates the significant influence of the monsoon season in Bangladesh, as the amount of rainfall closely corresponds to the normal patterns observed during the monsoon. The years 1999 and 2000 had the lowest recorded beginning rainfall levels, namely at 0.10 mm. Nevertheless, the amount of rainfall significantly rose throughout the course of the year, reaching 2172 mm and 2428 mm, respectively. This exemplifies the fluctuation and uncertainty of Bangladesh's yearly rainfall, since even years with minimal rainfall may ultimately experience a significant amount by the conclusion of the year. The dataset spanning 72 years of Bangladesh's annual rainfall levels exhibits a similar pattern of fluctuations, with certain years experiencing very high rainfall while others remain comparatively arid. This data underscores the substantial impact of the monsoon season on the rainfall patterns in the country, emphasizing the need of comprehending and managing these fluctuations in different sectors and aspects of life in Bangladesh.

#### 4.1.4 Mann-Kendall trend analysis

The Mann-Kendall test results, as shown in Table 2, offer crucial insights into the temporal patterns of the data set. Analysis of monthly and seasonal data reveals significant and noticeable patterns of change. Table 2 displayed a noteworthy negative Z value for the month of January, suggesting a possible decline in the variable being studied. This deviation from the average indicates a declining pattern. By contrast, the Z value for February is surprisingly close to zero, suggesting a minimal deviation from the average and therefore no noticeable pattern. In the future, March exhibits a negative sensitivity slope, suggesting a modest inclination towards a downward trend. This suggests that the variable is decreasing gradually, although the rate of change is not substantial. The S statistic, which quantifies the strength of a trend, indicates that December exhibits a highly significant positive value of 234. This signifies a notable upward trajectory, indicating that the variable being examined is increasing. The variable Var(s) offers information about the fluctuation of the Mann-Kendall statistic S. It indicates that winter is characterized by a high level of stability, with a value of 42314. This indicates that the S statistic remains constant over this period. As the study advances, the importance of the observed trends is increasingly established using *P*-values. Higher p-values, such as February's 0.9884, indicate a lack of statistically significant changes, while lower values, often below 0.05, suggest the presence of a noteworthy trend. As an illustration, the *P*-Value of 0.4338 in March signifies a noteworthy trend.

**Table 2:** Mann-Kendall test analysis.

Time	Z-Value	Sens Slope	S	Var(s)	*P*-Value	Tau
January	−1.7746	−0.08	−366	42303	0.076	−0.1432
February	−0.0146	0	−4	42306	0.9884	−0.0016
March	−0.7827	−0.149913	−162	42314	0.4338	0.4338
April	0.1458	0.081101	31	42315	0.884	0.0121
May	−0.0681	−0.052696	−15	42315	0.9457	−0.0059
June	−1.9251	−1.330458	−397	42315	0.0542	−0.1553
July	−0.8896	−0.462827	−184	42316	0.3737	−0.072
August	−0.7243	−0.348683	−150	42316	0.4689	−0.0587
September	−0.2139	−0.06956	−45	42315	0.8306	−0.0176
October	0.1021	0.063392	22	42314	0.9187	0.0086
November	−0.7487	−0.093472	−155	42313	0.4541	−0.0606
December	1.1331	0.017903	234	42287	0.2572	0.0915
Winter	−0.4813	−0.057944	−100	42314	0.6303	−0.0391
Pre-Monsoon	1.9883	1.580449	410	42316	0.0468	0.1604
Monsoon	−0.4521	−0.198958	−94	42316	0.6512	−0.0368
Post-Monsoon	−0.4521	−0.381864	−94	42316	0.6512	−0.0368
Annual	−1.9591	−3.68792	−404	42316	0.0501	−0.1581

The Tau-b statistic, commonly referred to as Tau, quantifies both the magnitude and direction of a monotonic trend. The Tau value for December was approximately 0.0915, suggesting a moderate upward trend. This indicates that, on average, the variable exhibits an upward trend over the course of this month. Nevertheless, it is crucial to situate these findings within the framework of the dataset and its domain. Factors such as the methodologies used for data collecting, external influences, and expertise in a specific field can offer valuable insights into the trends that have been seen. The Mann-Kendall test, a non-parametric statistical method, is employed to determine the Sen intensity steep and rainfall trend for each month over a span of 72 years, from January to December. The seasons that correlate to these are winter, pre-monsoon, monsoon, and post-monsoon. The trend of the 72-year series is demonstrated by the Z value statistic in the Mann-Kendall test for each of the twelve months from January to December, as well as for the winter, pre-monsoon, monsoon, and post-monsoon periods. The Z value exhibits a negative trend in January, February, March, May, June, July, and August, but it gradually rises in April, October, and December. The Z values for 8 months demonstrate a positive trend; however, the remaining 4 months exhibit a negative trend, indicating a state of insignificance. The pre-monsoon season exhibited a favorable seasonal pattern, but the remaining three seasons displayed unfavorable tendencies. The annual rainfall trend is decreasing. The Mann-Kendall test results provide a comprehensive understanding of the temporal trends in the dataset. Each season and month has its own distinct set of patterns, varying from minor to substantial alterations. Some time periods exhibit statistically significant alterations, while others remain relatively constant. Acquiring a comprehension of patterns in data sets is essential for making informed decisions and deriving valuable insights.

### 4.2 Spatial data analysis

This part presented and discussed the geographical data findings of the investigation. The geographical study, encompassing cartography and analysis of GIS data, unveiled crucial insights into the spatial distribution of groundwater quality concerns, rainfall trends, and prospective areas for rainwater harvesting throughout Bangladesh.

#### 4.2.1 Arsenic in groundwater

**Fig. 4:**
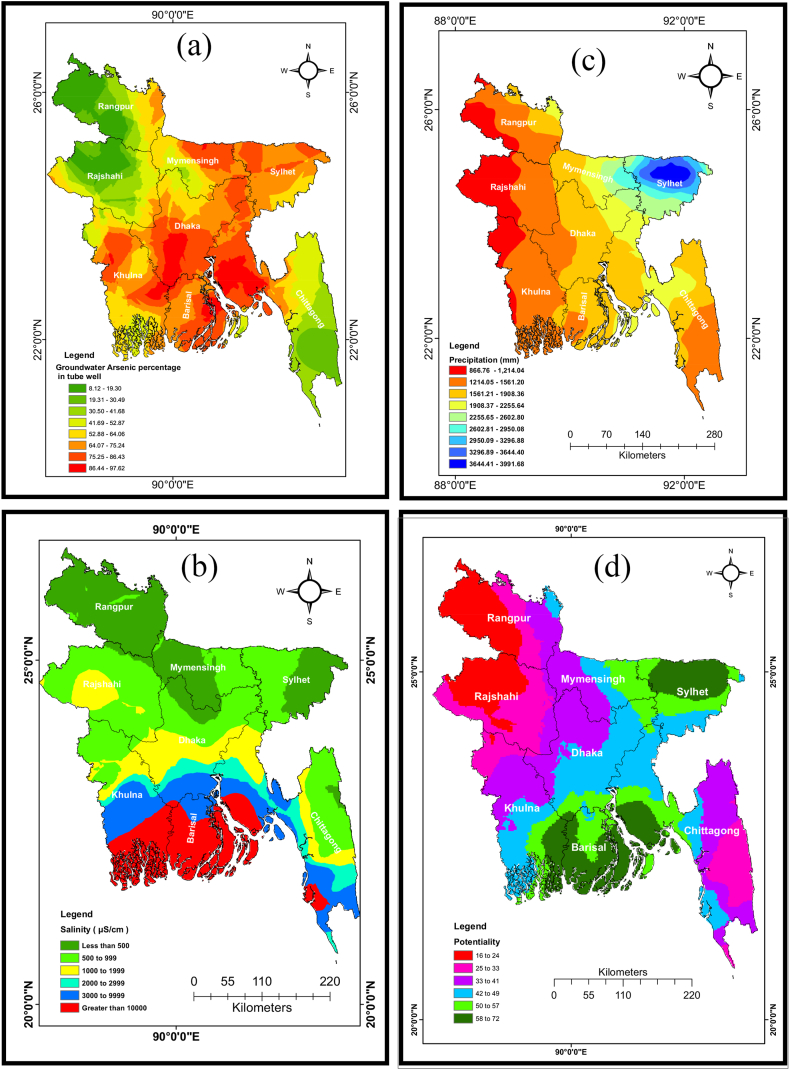
Spatial distribution of (a) Groundwater arsenic, (b) Groundwater salinity, (c) Average annual precipitation, and (d) Rainwater harvesting suitability zone of Bangladesh. Fig. 4 presents the spatial distribution of key environmental factors in Bangladesh. Panel (a) shows the spatial distribution of groundwater arsenic concentration. Panel (b) depicts the spatial distribution of groundwater salinity. Panel (c) illustrates the average annual rainfall across the country. Finally, panel (d) identifies rainwater harvesting suitability zones based on combined factors like rainfall and water quality.

Fig. 4(a) provides a representation of the geographical distribution of arsenic (As) in groundwater in Bangladesh. This figure helps us to comprehend the magnitude and spatial arrangement of this water quality problem. Arsenic contamination in tube well water is a significant issue in numerous regions of Bangladesh, exhibiting various levels of severity across different districts. The problem is highly significant in certain districts, like as Dhaka, Manikganj, Narshingdi, Munshiganj, Chandpur, Cumilla, and Lakshmipur, where over 80 % of tube wells exhibit heightened levels of arsenic. This critical situation necessitates prompt attention and remedial measures to guarantee that the impacted communities have access to uncontaminated drinking water. The percentage of arsenic pollution in tubewell water varied from 60 % to 80 % in the districts of Pirojpur, Jhalokathi, Patuakhali, Barguna, Khulna, and Bagerhat [Fig. 4(a)]. Arsenic pollution levels in certain areas of Chittagong, Satkhira, Sunamganj, Jessore, Faridpur, and Pabna districts range from 40 % to 60 %. Arsenic contamination levels ranging from 20 % to 40 % have been detected in the northern regions of the country, specifically in Kurigram, Netrokona, and Moulovibazar. However, the degree of pollution in these places is comparatively lower than in other regions. When comparing, the levels of arsenic contamination in the western and northeastern parts of Bangladesh, including Kishoreganj, Brahmanbaria, Chuadanga, Jhenaidah, Rajshahi, Natore, and other districts, are below 20 %.

#### 4.2.2 Salinity level in groundwater

The spatial distribution of salinity levels in Bangladesh offers insights on the extent of saltwater intrusion into groundwater and its regional variability. Given that the primary objective of this study is to address salinity problems, the data will be examined in the next section with regards to the possibility for collecting rainwater. Based on the data presented in Fig. 4(b), the results could assist in determining suitable sites for collecting and storing rainfall in regions affected by high levels of salt in the groundwater. The brackish waterways are predominantly located in the southern and southwestern sections of the country, including Shatkhira and Khulna districts. Furthermore, the findings indicate that saline water also impacts the south-central coastal area, encompassing Bagerhat, Pirojpur, Jhalokathi, Barisal, Barguna, Patuakhali, Bhola, Lakshmipur, Noakhali, and Chittagong.

#### 4.2.3 Variation of rainfall

The rainfall pattern in Bangladesh has provided insight into the spatial distribution of rainfall throughout the country. Fig. 4(c) displays the mean rainfall in multiple districts of Bangladesh from 1950 to 2023, demonstrating notable differences across different regions. The Sylhet division in the Northeast areas experiences the most substantial amount of rainfall, with rainfall levels ranging from 3644 mm to 3991 mm. This area encompasses the administrative divisions of Sylhet and Sunamganj. The annual rainfall in Moulovibazar varies between 3296 mm and 3644 mm. The amount of rainfall in Habiganj and Netrokona districts ranged from 2602 mm to 2950 mm and from 2950 mm to 3296 mm, respectively. Moulovibazar, Habiganj, Kishoreganj, and Netrokona, located in the southeast, experienced rainfall ranging from 2255 to 2602 mm. Meanwhile, in the eastern regions of Bangladesh, districts such as Narsingdi, Brahmanbaria, Feni, Mymensingh, Kurigram, Cumilla, and Khagrachari have had rainfall ranging from 1908 mm to 2255 mm. The annual rainfall varies between 1561 mm and 1908 mm, impacting the districts of Sherpur, Jamalpur, and Tangail in the northern region, as well as Patuakhali, Noakhali, and Bhola in the southern region. Additionally, the rainfall also affects Rangamati and Chittagong in the southeastern part of the country. The northwestern districts of Panchagarh, Nilphamari, and Rangpur, as well as the southern districts of Bagherhat and Khulna, and the western districts of Jessore, Narail, and Magura get relatively little rainfall, with amounts ranging from 1214 mm to 1561 mm. The western districts of Thakurgaon, Dinajpur, Naogaon, Nawabganj, Rajshahi, Kushtia, Meherpur, Chaudanga, and parts of Jaypurhat, Natore, and Shatkhira experienced the lowest amount of rainfall, ranging from 866 mm to 1214 mm. The range of rainfall in Bangladesh exhibits a four-fold difference between the lowest and highest values, highlighting the wide range of meteorological conditions seen throughout the country.

#### 4.2.4 Divisional precipitation, groundwater arsenic and groundwater salinity

The findings from the regional study of divisional rainfall, groundwater arsenic, and groundwater salinity in Bangladesh provide trustworthy data for enhancing rainwater collection. Consumers can acquire information about suitable locations for rainwater storage and the level of sustainability for water collection.

Fig. 4(c) displays the range of rainfall in the Chittagong division, which varied from 1214 mm to 2255 mm. Feni, Khagrachari, and Comilla experience higher levels of rainfall compared to the remaining districts of the Chittagong division, with rainfall ranging from 1908 mm to 2255 mm. The salinity levels in the Chittagong division vary from 500 μS/cm and 999 μS/cm, encompassing portions of the Rangamati and Khagrachari districts. The salt levels in the coastal parts of Chittagong division, such as Cox's Bazar and Noakhali, surpass 10,000 μS/cm, making them the highest in the region. Currently, there is significant apprehension regarding the presence of elevated amounts of arsenic in the groundwater of many districts (Comilla, Chandpur, Lakhshmipur, Noakhali, and Feni) within the Chittagong division.

The districts of Manikganj, Rajbari, and Faridpur in the Dhaka division see relatively low levels of rainfall, ranging from 1214 mm to 1561 mm [Fig. 4(c)]. When comparing them, Dhaka, Gazipur, Gopalganj, Madaripur, Narayanganj, Munshiganj, Shariatpur, and Tangail experience a moderate amount of rainfall, ranging from 1561 mm to 1908 mm. The districts of Kishoreganj and Narshingdi in the Dhaka division see the highest levels of rainfall, ranging from 1908 mm to 2255 mm. The salinity levels in the Dhaka division vary widely, ranging from less than 500 μS/cm to above 10,000 μS/cm. The salinity levels in Tangail district are the lowest. A significant presence of elevated levels of arsenic was detected in several regions of the Dhaka division, encompassing around 30 %–97 % of the entire area. Tangail district exhibits the lowest concentration of arsenic, whereas Gopalganj, Faridpur, and Kishorganj districts demonstrate the highest values, ranging from 75 % to 97 %.

The rainfall in Barishal Division varies between 1214 mm and 1561 mm, and between 1561 mm and 1908 mm, except for certain locations in Bhola, which experience rainfall ranging from 1908 mm to 2255 mm [Fig. 4(c)]. The rainfall in Barguna is comparatively lower than that in the neighbouring regions. The annual rainfall of Barisal, Bhola, Jhalokati, Pirojpur, and Patuakhali varies from 1561 mm to 1908 mm. The problem of high salt content in groundwater is a significant concern in the southern region of Bangladesh, particularly in the Barishal division. The salinity levels of Barishal district are significantly lower compared to other districts in the division. of fact, almost all other districts have salinity levels exceeding 10,000 μS/cm. Moreover, the concentration of arsenic in groundwater varies from 41 % to 97 % over most parts of Barishal.

Bagerhat, Jessore, Jhenaidah, Khulna, Magura, Narail, and Satkhira saw greater rainfall compared to the remaining districts in the Khulna division, with rainfall ranging from 1214 mm to 1561 mm as depicted in Fig. 4(c). Chuadanga, Kushtia, and Meherpur see a relatively low amount of rainfall, ranging from 866 mm to 1214 mm. The salinity gradient in the Khulna division exhibits a consistent increase from north to south. The districts of Khustia, Meherpur, Chuadanga, and Jhinaidaha exhibit the division's most minimal salinity levels, which are within the range of 500–999 μS/cm. The Satkhira, Khulna, and Bagerhat districts have the greatest salt levels, exceeding 10000 μS/cm. The concentration of arsenic in groundwater exhibits significant variation across the Khulna division, with levels ranging from 19 % to 97.5 %.

Based on the data presented in Fig. 4(c), Mymensingh and Netrokona experience higher levels of rainfall compared to Jamalpur and Sherpur. The recorded values range from 1561 mm to 1908 mm. The salt levels in Mymensingh division are among the lowest in Bangladesh. The salinity levels vary from below 500 μS/cm to 999 μS/cm, with the Netrokona district having the lowest salinity (below 500 μS/cm). Conversely, the Mymensingh division is troubled by the existence of arsenic in the underground water. The districts of Netrokona and Mymensingh exhibit the most elevated concentrations of arsenic, varying from 75 % to 97 %, while certain areas of Mymensingh, Sherpur, and Jamalpur display lower amounts, ranging from 30 % to 52 %.

The rainfall in Rajshahi division, as indicated by Fig. 4(c), varied between 3644 mm and 3991 mm. Additional districts seeing elevated levels of rainfall range from 3296 mm to 3644 mm and from 2950 mm to 3296 mm. All districts within the Rajshahi division exhibit a low level of salinity, with several regions in Bogura and Jaipurhat districts having salinities measuring below 500 μS/cm. The salinity levels in Rajshahi division range from 1000 μS/cm to 1999 μS/cm, with the maximum concentrations observed in the Rajshahi and Pabna districts. The levels of arsenic in nearly all districts in the Rajshahi division are relatively low, varying from 8 % to 75 %. The Naogan district exhibits the most minimal concentrations of arsenic, ranging from 8 % to 19 %.

The districts of the Rangpur division include Kurigram, Gaibandha, Thakurgaon, Dinajpur, Nilphamari, Panchagarh, Rangpur, and Lalmonirhat, as seen in Fig. 4(c). Except for Kurigram and Lalmonirhat, nearly every district in this division encounters negligible rainfall. The Rangpur division in Bangladesh has the lowest salinity levels in the country's groundwater. Each district in this division has a salinity level of less than 500 μS/cm. The arsenic concentrations in this region are very modest, varying from 8 % to 75 % of tubewells. Panchagarh, Thakurgaon, and Dinajpur exhibit the most minimal levels of arsenic, ranging from 8 % to 19 %. In contrast, the Kurigram district exhibits the highest concentrations of arsenic, ranging from 64 % to 75 %.

Fig. 4(c) illustrates the composition of the Sylhet division, which consists of four districts: Sylhet, Sunamganj, Moulvibazar, and Habiganj. Sunamganj and Sylhet districts receive a greater amount of rainfall compared to any other location in the country. The cumulative rainfall varies between 3644- and 3991-mm. Additional districts exhibit rainfall amounts ranging from 1971 mm to 3644 mm and 2950 mm–3296 mm. The salinity levels in nearly every district of Bangladesh range from 500 μS/cm to 999 μS/cm. The districts of Sylhet, Maulavibazar, Sunamganj, and Habiganj have the lowest salinity levels. Nevertheless, the levels of arsenic are elevated in nearly all districts within this division, with the most extreme amounts observed in Sylhet and Sunamganj, ranging from 75 % to 86 %.

#### 4.2.5 Rainwater harvesting suitable zone of Bangladesh

Rainwater harvesting is a straightforward and efficient method for addressing water-related challenges in Bangladesh, such as salinity, arsenic poisoning in groundwater, and climate change. Fig. 4(d) depicts a map of Bangladesh indicating the specific areas designated for rainwater gathering. The regions with the greatest suitability are situated in the eastern section of the country, specifically in the Brahmaputra River basin. The reason for this is that the eastern area of Bangladesh experiences the most amount of rainfall in the country. Based on the map, the areas in Bangladesh with the highest capacity for collecting rainwater include the Sylhet division, Mymensingh division, Rangpur division, the northern portion of Rajshahi division, the northern half of Dhaka division, and the southern section [Fig. 4(d)]. These locations are well-suited for collecting rainwater because of several aspects, such as abundant rainfall, mostly level land, appropriate soil types, the absence of saltiness and arsenic, and the availability of rooftops and other areas where rainwater can be collected. Bangladesh possesses a considerable capacity for the collection of rainwater. The country's northeast experiences the most amount of rainfall, and the south receives the lowest amount.

The northwestern part of Bangladesh is characterised by its arid climate, making it the least humid area in the country. The water has a very low concentration of arsenic and nearly little salinity. Consequently, the gathering of rainwater in this area is restricted. Central Bangladesh, including Dhaka, Manikganj, and Narshingdi, has more rainfall. Nevertheless, the levels of arsenic have increased in almost 80 % of the tube wells at this site. Implementing rainwater harvesting could address the region's arsenic issue.

The Sylhet division, located in the eastern region of Bangladesh, experiences the most abundant rainfall. The rainwater suitability in this region is the highest compared to other sections of the country. Conversely, the southern coastal region has elevated amounts of salt. In these places, rainwater collecting can serve as an alternative source of fresh water, despite the moderate suitable for rainfall. The northern Rajshahi region (Barin region) has a moderate suitable for rainwater collection due to water scarcity and contamination with As. Therefore, implementing rainwater gathering techniques could alleviate the water scarcity in this region. Rainwater harvesting in Bangladesh has significant suitability, yet its effectiveness varies depending on the region. The northern portion of the country has the lowest suitability, whilst the eastern and southern regions have the most suitable. The middle area of the country exhibits a moderate level of promise.

## 5 Discussion

Our results along with previous studies [5,[35], [36], [37]] indicated that the tubewells had arsenic concentrations higher than the worrying 0.01 mg/L WHO limit [38]. The concentration of arsenic rises as one goes deeper down a well [39]. Each district has a different level of the issue; in some, the contamination in tube wells is over 80 %. The districts with the most damage was Chandpur, Cumilla, Lakshmipur, Narshingdi, Munshiganj, Manikganj, and Dhaka. There have been reports of excessive arsenic levels in the districts of Bagerhat, Jhalokathi, Patuakhali, Barguna, Khulna, and Pirojpur. The problem of arsenic poisoning persists even in places where the frequency of cases is lower. Chittagong, Sunamganj, Jessore, Faridpur, and Pabna are among the impacted districts; the percentage of contamination in tube wells varies from 40 % to 60 %. With levels of less than 20 %, Bangladesh's northeastern and western regions are least contaminated. Our findings and those of other studies [8,9,[40], [41], [42]] suggest that the high salinity issue in southwest Bangladesh's groundwater, which is made worse by the infiltration of salty Bay of Bengal water, is comparable to a slow but persistent invasion on the nation's vital resources. Depending on the water's salinity, groundwater in southwest Bangladesh can have salinities ranging from 0.5 to 5.2 ppt. The EC ranges between 1 and 10 μS/cm. The Bangladeshi government's suggested salinity threshold of 2 μS/cm was surpassed in all groundwater testing [38]. The southern and southeast portions of the ideal region contain most of the sensitive zones, with the north being an especially vulnerable sector [43]. Bangladesh's coastline makes up about 53 % of its total land area, with 20 % of this area being influenced by varying salinities [44]. Bangladesh's groundwater salinity is a serious issue, especially in the southwest and south of the nation. The existence of salty water in the Bay of Bengal is the cause of this issue. The districts most impacted by salinity of groundwater are Shatkhira, Khulna, Bagerhat, Pirojpur, Jhalokathi, Barisal, Barguna, Patuakhali, Bhola, Lakshmipur, Noakhali, and Chittagong. In Bangladesh's southwest, the geochemical process of rock-water contact has the biggest impact on groundwater quality. Calcite crystals were broken down in this process, and then silicate minerals weathered [45,46]. The influx of salty groundwater poses significant challenges to sustainable agriculture, potable water supplies, and the environment.

Bangladesh's rainfall patterns provide a special difficulty for water management. Seventy-two years of research have shown that the nation is extremely susceptible to severe weather. The monsoon season, which lasts from May to October, accounts for more than 89 % of all annual precipitation, with June, July, and August receiving particularly high amounts [47,48]. The development of rainwater gathering systems benefits from the regularity of rainfall patterns. However, the employment of location-specific solutions is required due to the unequal distribution of rainfall across the nation, with most of the rain falling on the northeast and southeast coasts [[49], [50], [51]]. The significant variation in seasonal weather patterns highlights the necessity of rainwater collection to adapt to changing circumstances. After extended periods of low rainfall, rainwater collected during the monsoon season can be stored and used to meet water needs. RWH systems have claimed to save a significant amount of water, proving their economic viability [52]. Rainwater collecting might help centralized water reservoirs in Bangladesh's coastal areas become less vulnerable, as they are particularly vulnerable to natural catastrophes and climate change [53].

Collecting rainwater is an interesting way to control water supplies in Bangladesh, especially in places where problems like arsenic in the groundwater and saltwater getting into coastal areas are a problem [14,54]. Studies have looked at how well methods set up by NGOs and the government for collecting rainwater work [54]. These methods are mostly used for cooking and drinking in homes [54]. But there are some things that could be done better. One study found that home holding tanks don't always hold enough water to meet year-round needs, so many people use other water sources [55]. Also, people need to learn more about how to treat water and keep their rainwater collection methods in good shape [55]. Another study talks about the benefits of systems that collect rainwater after disasters like Cyclone Aila. These systems were very important for getting clean drinking water after traditional sources were damaged [14]. But problems like the distance to fetching points and making sure they are open all year remain [14].

In many countries, collecting rainwater offers a sustainable solution. It is essential for rural development and is commonly utilized in Indian agriculture [56,57]. The effect of rainwater collection on urban water systems is advantageous to Germany [58]. Brazil noted that collecting rainwater is advantageous for sustaining urban gardens, irrigating university landscapes, and lowering the burden on the water supply [59]. Some Asian nations, like South Korea, benefit from less environmental impact on water systems [60], while Singapore and Japan both use treated wastewater to recharge groundwater [61,62]. In Pakistan, rainwater collection is used to store monsoon rainwater for future consumption and to recharge aquifers [63].

From the literatures, we found that rainwater collection has some disadvantages, such as the fact that it frequently results in undesirable byproducts, the need for a large storage tank, and the application of insufficient disinfection treatments. Moreover, there are significant concentrations of iron (Fe), copper (Cu), zinc (Zn), and manganese in rainwater that may have been contaminated by the air. The water had to be treated before it could be drunk because the pH, nitrate, and fluoride values in Dhaka city differed significantly from the norms [[64], [65], [66]].

Long-term storage remains a difficulty, even with cleaner rainwater. Here, the First Flush Diverter is a useful instrument to employ. The first surge of roof runoff, which is usually accompanied by dirt, leaves, and other surface material, is diverted by this device. It is possible to improve the collected water quality by transferring contaminated water to a different, cleaner source and away from the storage tank [55]. It is plausible that employing this recently developed initial rainfall harvesting technology for rainwater collection could be a wise, advantageous, and economical strategy to stop surface and groundwater depletion in Dhaka based on a thorough analysis of the financial and practical viability of first-flush technology [67]. It is important to emphasize that whereas a large home can only partially achieve water supply reliability, a small household can achieve maximum reliability. Rainwater naturally contains no minerals and is soft, but when it is stored, it can get contaminated with bacteria and other microorganisms [68]. These results highlight the need for suitable legislation and RWH system treatment procedures when used for non-potable uses [69,70]. German specialists claim that because of microbial hazards, collected rainwater may need to be treated before being used for non-potable purposes [58]. More control over the bacterial community could improve the quality of tank water, according to a Chinese study [71]. Microbial populations in rainwater storage tanks need to be carefully controlled to maintain water quality and prevent the development of opportunistic diseases [72]. Rainwater can be treated with household filtering devices to make it safe to drink. Using a ceramic pot filter or a slow sand filter is an additional choice; these two types of filters provide physical barriers that can hold bacteria, parasites, and other dangerous microorganisms [73].

There are many errors in the study on rainwater collection in Bangladesh. Our limited access to secondary data sources forms the basis of this investigation. This suggests that the limits of secondary data constrain the study's conclusions. For instance, the study's conclusions will be inaccurate or lacking if the secondary data is inaccurate or nonexistent. Notwithstanding these limitations, the study provides valuable and novel insights about Bangladesh's capacity to harvest precipitation runoff. The results of the study can be utilized to customise rainwater collection initiatives to Bangladesh's unique suitability. One potential solution to Bangladesh's water problems is to widely install rainwater collection devices. But for implementation to be successful, giving communities and people financial and technical support is essential. In Bangladesh, communities, NGOs, and governments may all contribute to the development of rainwater harvesting. By putting these strategies into practice, stakeholders may actively encourage rainwater collecting in Bangladesh and profit from all its many advantages.

Despite these obstacles, research points to viable methods for expanding the use of rainwater collection. Particularly in rural regions, public awareness campaigns, instructional seminars, and tax breaks have the potential to greatly enhance adoption of RWH technology [74]. Studies have indicated that replenishing treated wastewater into aquifers can raise water levels and enhance groundwater quality [75]. Hydrological model performance can be enhanced and water tank construction can be made more efficient with the use of remote sensing techniques [76]. RWH not only improves the quality of the water but also has positive environmental effects. Ozone depletion, ionising radiation, and water use have all decreased, per the research [77]. Research indicates that collected rainwater can be utilized for irrigation safely, despite concerns regarding the acceptability of rainfall for irrigation due to its salt concentration [78,79]. The study highlights the importance of implementing a holistic strategy in Bangladesh because of the intricate interactions that exist between salinity, arsenic toxicity, and climate change. Effective management of water resources in the face of environmental change depends on the confluence of scientific knowledge and workable solutions.

## 6 Conclusions

The study revealed that the groundwater of the southwest regions of Bangladesh (Khulna, Bagerhat, Satkhira, Barisal, Noakhali, and Bhola) were vulnerable to salinity contamination (>10000 μS/cm). Besides, the groundwater of the northeast regions (Sylhet and Cumilla) was contaminated with high concentration of arsenic. Most of the cases, arsenic was the prevalent contaminant in each of these regions. The findings from climate data indicated that the Sylhet region had the most suitable for rainwater collection, since it experienced the highest levels of rainfall (ranging from 2600 to 3900 mm). Barisal, Patuakhali, Borguna, Bhola, Noakhali, and Cumilla regions, on the other hand, had intense rainfall. Considering pollution level and rainfall availability, the integrated study from GIS method showed that the most appropriate and enduring regions for rainwater collection were Sylhet, Bagerhat, Barishal, Bhola, Noakhali, and Cumilla. The policy maker might consider the findings from this study when making decisions on the long-term secured supply of safe drinking water for the vulnerable community.

## Data availability statement

The spatial and temporal data analyzed during this current research are publicly available in a dedicated database. The data can be accessed directly through the following link: https://doi.org/10.5281/zenodo.12210267.

Additionally, the spatial data is formatted to be compatible with Geographic Information System (GIS) software, including ArcGIS and QGIS. Users can download the data from the database and import it directly into their preferred GIS software for further analysis or visualization.

## CRediT authorship contribution statement

**Md. Abdullah:** Writing – review & editing, Writing – original draft, Visualization, Software, Resources, Project administration, Methodology, Investigation, Formal analysis, Conceptualization. **Fatin Idrak:** Writing – review & editing. **Purnima Kabir:** Writing – review & editing. **Mohammad Amir Hossain Bhuiyan:** Writing – review & editing, Supervision, Project administration, Investigation, Conceptualization.

## Declaration of competing interest

The authors declare that they have no known competing financial interests or personal relationships that could have appeared to influence the work reported in this paper.
